# Prevalence and determinants of cardiovascular disease risk factors among the residents of urban community housing projects in Malaysia

**DOI:** 10.1186/1471-2458-14-S3-S3

**Published:** 2014-11-24

**Authors:** Mohammadreza Amiri, Hazreen Abdul Majid, FarizahMohd Hairi, Nithiah Thangiah, Awang Bulgiba, Tin Tin Su

**Affiliations:** 1 Department of Development Studies, Faculty of Economics and Administration, University of Malaya, 50603, Kuala Lumpur, Malaysia; 2 Centre for Population Health (CePH), Department of Social and Preventive Medicine, Faculty of Medicine, University of Malaya, 50603, Kuala Lumpur, Malaysia; 3 Julius Centre University of Malaya (JCUM), Department of Social and Preventive Medicine, Faculty of Medicine, University of Malaya, 50603, Kuala Lumpur, Malaysia

**Keywords:** Cardiovascular Disease Risk Factors, Prevalence, Determinants, Socioeconomic Status, Low Income, Malaysia

## Abstract

**Objectives:**

The objectives are to assess the prevalence and determinants of cardiovascular disease (CVD) risk factors among the residents of Community Housing Projects in metropolitan Kuala Lumpur, Malaysia.

**Method:**

By using simple random sampling, we selected and surveyed 833 households which comprised of 3,722 individuals. Out of the 2,360 adults, 50.5% participated in blood sampling and anthropometric measurement sessions. Uni and bivariate data analysis and multivariate binary logistic regression were applied to identify demographic and socioeconomic determinants of the existence of having at least one CVD risk factor.

**Results:**

As a Result, while obesity (54.8%), hypercholesterolemia (51.5%), and hypertension (39.3%) were the most common CVD risk factors among the low-income respondents, smoking (16.3%), diabetes mellitus (7.8%) and alcohol consumption (1.4%) were the least prevalent. Finally, the results from the multivariate binary logistic model illustrated that compared to the Malays, the Indians were 41% less likely to have at least one of the CVD risk factors (OR = 0.59; 95% CI: 0.37 - 0.93).

**Conclusion:**

In Conclusion, the low-income individuals were at higher risk of developing CVDs. Prospective policies addressing preventive actions and increased awareness focusing on low-income communities are highly recommended and to consider age, gender, ethnic backgrounds, and occupation classes.

## Background

While declining trends in mortality due to CVD have been observed in developed nations in the recent decades, there has been a dramatic rise in low and middle-income countries instead [1,2]. As the transition towards developed economies raise living standards, it may jeopardize the health of lower socioeconomic groups. In addition, economic growth and urbanization are associated with higher prevalence of chronic non-communicable diseases (NCD) in developing countries [3]. Hence, population in developing nations is at a higher risk of getting CVDs [4-6].

Malaysia is classified as a developing country [7]. It is a multi-ethnic nation with a growing population[8] experiencing rapid urbanization and vast changes in lifestyles, including poorer dietary habits and less physical activities [9]. Chronic illnesses in Malaysia are due to both demographic and socioeconomic transitions caused by economic development [10] which will result in higher prevalence of NCDs in Malaysia [11]. Mortality due to CVDs in Malaysian hospitals has risen dramatically from 15.7% in 1996 to 25.4% in 2006 [12,13] and is expected to increase in the subsequent decades [14,15].

Malaysia consists of different ethnic and socioeconomic groups [8,16]. It is proven that socioeconomic status (SES) influences the health condition [17-19]. In addition, higher socioeconomic inequalities [20] and lower levels of income [21], education [22,23], and occupational status [2,24] are highly associated with the risk of CVDs. Furthermore, living in urban areas increases the risk of CVDs [25] as the health expenditures are higher, socioeconomic inequalities are wider, and the living environment is worse compared to rural areas. Finally, besides environmental transition, urban transition also influences the living lifestyle, including living costs, higher stress level, dietary habits, physical activities, and smoking habits, which are more damaging to the health condition [26].

In this study, we aimed at identifying the prevalence and determinants of the most established CVD risk factors (i.e. hypertension, hypercholesterolemia, smoking, overweight and/or obesity [27-29] and diabetes mellitus [30,31]) among the residents of urban Community Housing Projects [i.e. in local language: PPR (Projek Perumahan Rakyat)] located in the metropolitan Kuala Lumpur, Malaysia. These Community Housing Projects are densely populated with low socioeconomic groups. This study has several advantages compared to the previous research. First, it is an unprecedented research in studying the PPR residents in Malaysian's metropolitan areas. Second, this study not only covers as many CVD risk factors as the previous nation-wide studies have done [12-15], but in fact it has more risk factors compared to them [32-35]. Finally, this study is the baseline for a-five-year longitudinal research and the findings from this study will be applied to establish tailor-made interventions for vulnerable lower socioeconomic population in metropolitan areas.

## Methods

### Study setting

The study was conducted in four Community Housing Projects [i.e. PPR Kerinchi, PPR Pantai Ria, PPR Seri Cempaka and PPR Seri Pantai] located in the Lembah Pantai area of metropolitan Kuala Lumpur, Malaysia. These PPRs were developed by Kuala Lumpur City Hall in their squatter resettlement programme. The units in the community housing projects were only allocated to families: 1.with at least one child; 2.with household's monthly income below MYR 2,000 (MYR: Malaysian Ringgit); and, 3.who does not own any property within 35 km from Kuala Lumpur. These requirements were applied in the early 2000 when the resettlement programme was established.

### Data collection and sample size

Sample size was determined by Stata software v11.2 (StataCorp LP, TX, USA). For the population, we used standardised population of Malaysia who live in the urban areas (i.e. about 20 million individuals, which is 72% of the total population of Malaysia)[8]. Based on recent national survey [14], we used the highest CVD risk factors frequency in Malaysia [i.e. hypercholesterolemia (35.1%)]. We assumed the true frequencies of the surveyed population to lie between ± 5 percent confidence limits, the power to be 80%, and the confidence interval to be 95%. The calculated sample size was 350. However, we decided to include all eligible participants from the household survey conducted in Lembah Pantai area to increase the precision of the analysis.

This survey was a cross-sectional design and conducted between February to November 2012. Altogether, 833 households were recruited from the total of 4726 households from four Community Housing Projects by using simple random sampling method.

The members of the selected households were administered questionnaire survey in either Bahasa Malaysia or English. Then, the respondents aged eighteen years and above, were invited to the predefined medical centers for blood sampling and anthropometrical measurements. All evaluations were conducted by professional teams led by a medical doctor. Written consents were taken from all respondents.

### Measurement

Anthropometric measurements included height (measured by SECA 217 Stadiometer for Mobile Height Measurement and rounded to the nearest one mm), weight (measured by SECA 813 Digital High Capacity Floor Scale and rounded to the nearest 0.1 kg), and waist and hip circumference (measured by SECA 201Ergonomic circumference measuring tape in cm). The arterial blood pressure (measured by Omron HEM7211 Automatic Blood Pressure Monitor), and non-fasting random blood sugar and lipid profile were also assessed (by Dimension Vista^® ^1500 Intelligent Lab). The lab investigations were done in a certified laboratory of a tertiary hospital.

### Study variables

#### Cardiovascular disease risk factors

The CVD risk factors were classified as follows [27,36]: the elevated blood pressure was considered as hypertension if Systolic and/or Diastolic Blood Pressure equaled or above140/90 mm Hg or if any antihypertensive medications were used. To be a diabetic the Random Blood Sugar (RBS) readings were equal to or above 11.0 mmol/L and/or were under diabetes treatment [37]. In addition, high risk of diabetes was determined if the RBS levels were between 5.6 and 11.0 mmol/L. Those who had Total Cholesterol (TC) ≥5.2 mmol/L and/or were using cholesterol-lowering drugs were categorized as hypercholesterolemics. To qualify as overweight and obese, the Asian classifications of Body Mass Index (BMI, kg/m^2^)from 23.0 to 24.9, and over 25.0 kg/m^2 ^were used respectively [38,39]. However, there have been debates on consideration of 25 kg/m^2 ^up to 27.5 kg/m^2 ^as overweight or pre-obese for Asians [38-42], but we chose to classify 25 kg/m^2 ^and above (i.e. where public health interventions must take effect) as obesity (i.e. obesity includes high risk overweight, pre-obese and obese types I, II, and III) according to WHO Expert Consultation [38]. Smokers were adults who smoked at least a cigarette per day[43]. Drinking one sip of alcoholic beverage per day was considered as alcohol risk factor[1]. Having at least one of the above CVD risk factors was considered as the outcome of the logistic regression model.

#### Demographic and socioeconomic characteristics

Age classifications were 18 to 29, 30 to 39, 40 to 49, 50 to 59, and 60≤ in the bivariate analysis. However, the logistic model was controlled for continuous values of age. Ethnically, the population distribution of Malaysia is Malays (67.4%), Chinese (24.6%), Indians (7.3%), and other ethnic/race groups (0.7%) [8]. Hence, we classified the ethnic/race variable into Malays, Indians, and Chinese or others minor ethnic groups. Finally, the marital status was categorized as single, married, widowed/widower, and divorced sub-groups[16].

The education grades were classified as none (zero years of study), primary (<7 years), secondary (7-12 years) and tertiary levels (>12 years). For classification of income levels, the Malaysia's cut-off point of poverty line in the year 2009 (i.e. MYR786) was considered and adjusted for inflation rate and living expenses of metropolitan Kuala Lumpur [44]. Hence, after rounding up, MYR1,000 was the threshold of poverty. Therefore, the monthly household income was classified as MYR<1,000 (the poor) or others. Finally, the paid-employee, self-employed, retirees, homemakers, and others were the categories of occupational status.

### Statistical analyses

First, descriptive data analysis was carried out. Then, individual and existence of at least the CVD risk factors were tabulated against the demographic and socioeconomic variables. The χ^2 ^probabilityillustrated whether the relationships were significant or not (note: level of 0.05 was considered). Finally, binomial multivariate logistic regression assessed the demographic and socioeconomic determinants of the existence of at least one of the CVD risk factors. We followed the STROBE statement in reporting our research [45].

## Results and discussion

### Descriptive analysis

Altogether, the data from 3,722 individuals (833 households) were collected in the initial household survey in the PPRs. Then, from 2,360 respondents (≥18 years old) who were invited to attend the medical screening and anthropometric measurements, 1,192 (50.5%) participated. We further tested the representativeness of the selected respondents according to age distribution, gender, and ethnic/race groups and we saw no significant difference existed.

We had comprehensive data for 1,096 respondents after eliminating the missing values. The mean age of the respondents was 41.5 years (SD: 14.9). The majority of the respondents were over 30 years old (73.8%), females (56.3%), Malays (82.1%), married (64.0%), and had secondary education (65.5%). Less than MYR1,000 per month earners were 17.5 percent of the whole sample population (see Table 1).

**Table 1:** Socio-demographic characteristics of the study (n = 1,096).

Variables	Sub-categories	N (%)
**Age**	*18 to 29*	288 (26.3)
	*30 to 39*	213 (19.4)
	*40 to 49*	255 (23.3)
	*50 to 59*	201 (18.4)
	*≥ 60*	139 (12.7)
**Gender**	*Male*	479 (43.7)
	*Female*	517 (56.3)
**Ethnicity**	*Malay*	899 (82.1)
	*Indian*	181 (16.5)
	*Chinese/Others*	16 (1.4)
**Education**	*None*	78 (7.1)
	*Primary*	186 (17.0)
	*Secondary*	718 (65.5)
	*Tertiary*	114 (10.4)
**Income**	*< 1,000 RM*	192 (17.5)
	*≥ 1,000 RM*	904 (82.5)
**Occupation**	*Paid-employee*	463 (42.2)
	*Self-employed*	138 (12.6)
	*Retiree*	42 (3.8)
	*House maker*	237 (21.6)
	*Others*	216 (19.7)
**Marital status**	*Single*	277 (25.3)
	*Married*	701 (64.0)
	*Divorced*	67 (6.1)
	*Widow/Widower*	51 (4.6)

Obesity (54.8%), hypercholesterolemia (51.5%), and hypertension (39.3%) were the most prevalent CVD risk factors. According to The Fourth National Health and Morbidity Survey of Malaysia [14], the obesity, hypercholesterolemia and hypertension risk factors in Malaysia accounted for 27.2, 35.1, and 32.7 percent respectively. Hence, comparing the figures between the low-income urban communities and at the national level, the prevalence of each CVD risk factor is much higher in the low-income community group. For instance, the low-income adults were more than two times obese compared to the national level. Finally, the highest prevalence of at least one of the CVD risk factors was among 50 to 59 year-old adults, male, Chinese, and divorced (demographically), and the primary educated, less than MYR1,000 monthly income earners, and self-employed adults (socioeconomically).

Demographically, respondents in their 50's and above 60 years had the highest prevalence of hypertension (i.e. 63.7 and 73.4 percent respectively) and diabetes (i.e. 17.9 and 11.5). Hypercholesterolemia and obesity were the highest among middle-aged adults who were in their 40's (i.e. 62.4% and 64.3% respectively). Younger respondents had higher prevalence of smoking, i.e. 30's and 40's accounted for 21.1 and 19.2 percent respectively. The widowed and divorced were most hypertensive. In addition, diabetes and obesity were the most prevalent in divorced people with 13.4 and 67.2 percent respectively. Those who were married had the highest prevalence of hypercholesterolemia and smoking, i.e. 57.6 and 19.5 percent respectively. Respondents with primary education had the highest level of hypertension (58.6%), diabetes (11.8%), and obesity (60.2%) whereas adults with secondary education had the highest prevalence of hypercholesterolemia and smoking by 53.8 and 17.6 percent respectively. In terms of occupational status, the highest prevalence of hypercholesterolemia, smoking, and diabetes were experienced by self-employed respondents. Finally, while the poorest respondents (i.e. <MYR1,000 monthly income) were the most hypertensive (50.5%) and being smokers (17.2%), the other income categories had higher prevalence of other CVD risk factors.(see Table 2).

**Table 2:** Prevalence of individual CVD risk factors (n = 1,096).

	Hypertension	Hypercholesterolemia	Smoking	Diabetes		Obesity		At least one
				**High Risk**	**Diabetic**	**Overweight**	**Obese**	

**Age**								
***18 to 29***	45 (15.6)	110 (38.2)	35 (12.2)	59 (20.5)	6 (2.1)	28 (9.7)	113 (39.2)	185 (64.2)
***30 to 39***	47 (22.1)	100 (46.9)	45 (21.1)	79 (37.1)	11 (5.2)	39 (18.3)	111 (52.1)	181 (85.0)
***40 to 49***	109 (42.8)	159 (62.4)	49 (19.2)	109 (42.8)	17 (6.7)	38 (14.9)	164 (64.3)	232 (91.0)
***50 to 59***	128 (63.7)	124 (61.7)	28 (13.9)	93 (46.3)	36 (17.9)	31 (15.4)	128 (63.7)	195 (97.0)
***> 60***	102 (73.4)	71 (51.1)	22 (15.8)	76 (54.7)	16 (11.5)	22 (15.8)	84 (60.4)	133 (95.7)
***prob***.	**<0.0001**	**<0.0001**	**<0.0001**	**<0.0001**		**<0.0001**		**<0.0001**
**Education**								
***None***	42 (53.9)	39 (50.0)	11 (14.1)	33 (42.3)	6 (7.7)	6 (7.7)	43 (55.1)	67 (85.9)
***Primary***	109 (58.6)	92 (49.5)	28 (15.1)	80 (43.0)	22 (11.8)	33 (17.7)	112 (60.2)	168 (90.3)
***Secondary***	261 (36.4)	386 (53.8)	126 (17.6)	267 (37.2)	53 (7.4)	105 (14.6)	389 (54.2)	610 (85.0)
***Tertiary***	19 (16.7)	47 (41.2)	14 (12.3)	36 (31.6)	5 (4.4)	14 (12.3)	56 (49.1)	84 (71.1)
***prob***.	**<0.0001**	**>0.05**	**>0.1**	**<0.05**		**<0.05**		**<0.0001**
**Income**								
***< 1,000***	97 (50.5)	96 (50.0)	33 (17.2)	77 (40.1)	12 (6.3)	26 (13.5)	95 (49.5)	169 (88.0)
***≥ 1,000***	334 (36.9)	468 (51.8)	146 (16.2)	339 (37.5)	74 (8.2)	132 (14.6)	505 (55.9)	757 (83.7)
***prob***.	**<0.001**	**>0.1**	**>0.1**	**>0.1**		**>0.1**		**>0.1**
**Occupation**								
***Paid-employee***	137 (29.6)	238 (51.4)	98 (21.2)	171 (36.9)	27 (5.8)	77 (16.6)	250 (54.0)	389 (84.0)
***Self-employed***	63 (45.7)	82 (59.4)	39 (28.3)	60 (43.5)	18 (13.0)	15 (10.9)	84 (60.9)	127 (92.0)
***Retiree***	30 (71.4)	19 (45.2)	11 (26.2)	23 (54.8)	4 (9.5)	7 (16.7)	25 (59.5)	38 (90.5)
***House maker***	113 (47.7)	136 (57.4)	3 (1.3)	92 (38.8)	21 (8.9)	34 (14.4)	143 (60.3)	208 (87.8)
***Others***	88 (40.7)	89 (41.2)	28 (13.0)	70 (32.4)	16 (17.4)	25 (11.6)	98 (45.4)	164 (75.9)
***prob***.	**<0.0001**	**<0.01**	**<0.0001**	**<0.01**		**<0.01**		**<0.0001**
**Marital status**								
***Single***	61 (22.0)	100 (36.1)	35 (12.6)	65 (23.5)	9 (3.3)	25 (9.0)	115 (41.5)	186 (67.2)
***Married***	300 (42.8)	404 (57.6)	137 (19.5)	298 (42.5)	62 (8.8)	115 (16.4)	413 (58.9)	632 (90.2)
***Divorced***	36 (53.7)	35 (52.2)	6 (9.0)	30 (44.8)	9 (13.4)	8 (11.9)	45 (67.2)	63 (94.0)
***Widow/Widower***	34 (66.7)	25 (49.0)	1 (2.0)	23 (45.1)	6 (11.8)	10 (19.6)	27 (52.9)	45 (88.2)
***prob***.	**<0.0001**	**<0.0001**	**<0.0001**	**<0.0001**		**<0.0001**		**<0.0001**
Total	431 (39.3)	564 (51.5)	179 (16.3)	416 (38.0)	86 (7.8)	158 (14.4)	600 (54.8)	926 (84.5)

**Figure 1:**
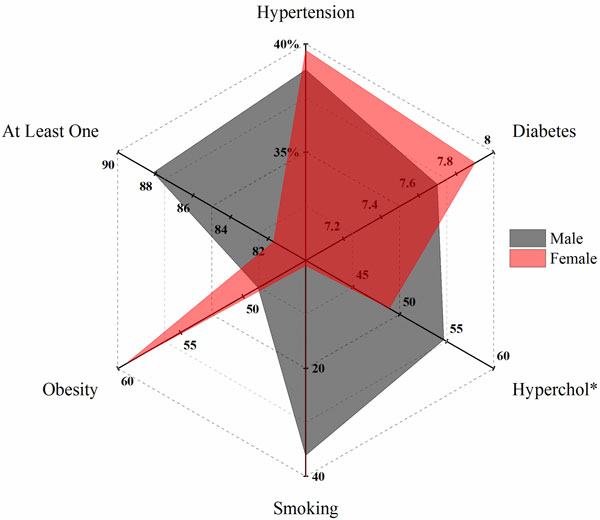
Prevalence of CVD risk factors by gender (to be under the image): *Hypercholesterolemia.

The prevalence of CVD risk factors according to gender is shown in Figure 1. For instance, comparatively men had a higher prevalence of hypercholesterolemia (54.7%) and smoking (36.1%), while women were more hypertensive (39.7%), diabetic (7.9%), and obese (59.3%).

**Figure 2:**
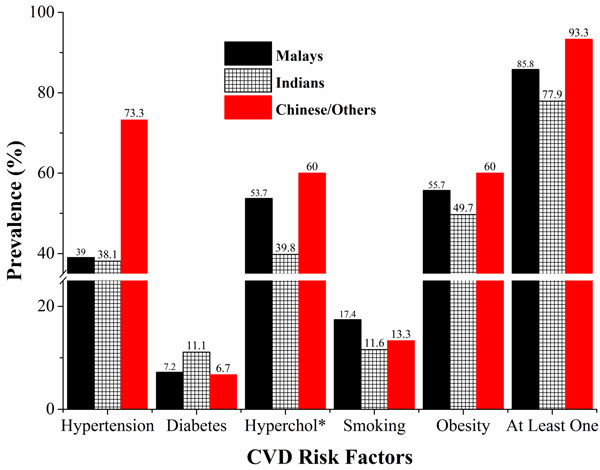
Ethnic differences in prevalence of CVD risk factors (to be under the image): *Hypercholesterolemia.

Figure 2 illustrates that all CVD risk factors were lowest among Indians except diabetes in which they had the highest prevalence (11.1% comparing to 7.2 and 6.7 percent in Malays and Chinese respectively) (see Figure 2).

### Logistic regression analysis

Crude and adjusted results of multivariate binary logistic regressions identified the significant determinants of the existence of at least one of the CVD risk factors in the low-income communities. The multivariate model showed that Indians were 41% less probable to have at least one of the CVD risk factors (OR = 0.59; 95% CI: 0.37 - 0.93) as compared to the Malay ethnic group. Occupational categories illustrated that the paid-employees were two times more likely to have more than one risk factor as compared to the reference group (OR = 2.09; 95% CI: 1.12 - 3.89) (see Table 3).

**Table 3:** Logistic regression results of the cumulative^1 ^CVD risk factor

	Adjusted	Crude
	**OR^2 ^(CI^3^)**	**OR(CI)**

**Age**	1.08*** (1.05 - 1.10)	1.08*** (1.06 - 1.10)
**Gender**		
***Female***	0.49** (0.32 - 0.75)	0.60** (0.42 - 0.84)
***Male***	Reference	Reference
**Ethnicity**		
***Indian***	0.59* (0.37 - 0.93)	0.58** (0.39 - 0.87)
***Chinese/Others***	1.21 (0.13 - 10.71)	2.34 (0.30 - 17.96)
***Malay***	Reference	Reference
**Education**		
***Primary***	1.18 (0.44 - 3.18)	1.53 (0.68 - 3.41)
***Secondary***	1.90 (0.78 - 4.63)	0.92 (0.47 - 1.81)
***Tertiary***	1.92 (0.72 - 5.11)	0.40* (0.18 - 0.85)
***None***	Reference	Reference
**Income**		
***≥ 1,000RM***	0.81 (0.47 - 1.39)	0.70 (0.43 - 1.12)
***< 1,000 RM***	Reference	Reference
**Occupation**		
***Retiree***	1.99 (0.89 - 4.45)	9.89*** (4.97 - 19.66)
***House maker***	2.13 (0.94 - 4.82)	8.04*** (4.37 - 14.78)
***Paid-employee***	2.10* (1.13 - 3.91)	5.89*** (3.46 - 10.02)
***Self-employed***	2.12 (0.86 - 5.21)	12.94*** (5.96 - 28.08)
***Others***	Reference	Reference
**Marital Status**		
***Single***	1.93 (0.60 - 6.16)	0.27** (0.11 - 0.66)
***Married***	2.50 (0.89 - 7.05)	1.22 (0.50 - 2.96)
***Divorced***	3.09 (0.77 - 12.45)	2.09 (0.56 - 7.87)
***Widow/Widower***	Reference	Reference

Note: 1. Existence of at least one CVD risk factor 2. Odds-Ratios 3. Confidence Interval (95%)

*. P < 0.05 **. P < 0.01 ***. P < 0.0001

While a few contrasts were revealed, our study was generally in line with the literature. The prevalence of hypertension was increasing with age [15,33]. A previous study showed that in Malaysia, the prevalence of hypercholesterolemia was directly related to ageing, and this tallied with our findings as well [46]; however, our findings contradicted with a previous finding in which a greater proportion of females suffered from hypercholesterolemia[15]. Males smoked more than females in our study respondents. It is in line with previous studies, but the prevalence of smoking was even higher than the national average [14,47]. It was previously shown that Malays shared the highest prevalence of CVD risk factors except diabetes, in which Indians had the highest dominance [10,48,49]. Our study supported these findings that the low-income Indians were more diabetic, and the prevalence of diabetic adults was approximately the national levels as reported formerly [14]. However, the results did not show significant difference in the prevalence of CVD risk factors among all ethnic groups except in hypercholesterolemia (p < 0.01) and hypertension (p < 0.05) risk factors.

Furthermore, our study supported previous findings in which high-income adults were more obese [32,50,51]. It nevertheless contradicted with the overweight category in which lower educated respondents had higher prevalence of being overweight [32]. In addition, lower grades of education were related to higher prevalence of hypertension [9,10,33]. Besides, the poorer adults had higher prevalence of the presence of at least one of the CVD risk factors [52]. We additionally found that in the low-income urban communities, the self-employed adults had the highest prevalence of hypercholesterolemia, smoking, obesity, and at least one of the CVD risk factors, which may be due to stressful working environment. Considering marital status, we found it is related to all CVD risk factors including overweight/obesity [53] and smoking [43].

Results from the binary logistic regressions identified that age [9,48] and gender [9,10] were significant determinants of the existence of at least one of the CVD risk factors in the low-income community. Finally, the self-employed and retired adults had the highest likelihood of having at least one of the CVD risk factors compared to the other groups. However, the results from multivariate logistic regressions indicated that compared to Malays, the Indians had the lowest likelihood of having at least one of the CVD risk factors[15], while the paid-employee category was the most significant category in occupational status.

## Conclusion

The low-income and the poor communities in Malaysia had higher prevalence of CVD risk factors compared to the recent epidemiologic research [14,15]. Specifically, Malays, compared to Indians; and paid-employees compared to the other occupation categories, experienced the highest prevalence of CVD risk factors. This high prevalence may be as a consequence of delays and/or not seeking appropriate treatments, unhealthy dietary habits and physical inactivity of these groups [15]. In addition, the occupational stress may be the other cause for the high prevalence of CVD risk factors (specifically smoking) among the paid-employees in the low-income communities of Malaysia [54]. Therefore, ethnic backgrounds and occupational status of the low-income communities must be considered for the prospective public health policies and interventions.

Our research had a few limitations which should be prevented in future studies. First, defining diabetes would be more reliable if the fasting blood samples were collected. Second, alcohol consumption data in Malaysia is quite limited due to social and religious aspects. Besides, since the number of Chinese, Indians, and other ethnic groups were too low, we could not present rough estimates of these ethnic groups specifically. Finally, our study was not a representative of the national low-income Malaysians but it broadly illustrated the low-income urban population of Malaysia.

Nonetheless, the CVD risk factors in the low-income and the poor Malaysians in the urban community were quite obvious in this study. Hence, a thorough attention is needed in future policies to reduce CVD risk factors among the low-income communities.

## List of abbreviations

CVD: Cardiovascular Disease; SES: Socioeconomic Status; NCD: Non-Communicable Disease; PPR: ProjekPerumahan Rakyat (Community Housing Project); RBS: Random Blood Sugar; BMI: Body Mass Index; MYR: Malaysian Ringgit; PLI: Poverty Line Income; OR: Odds-Ratio; CI: Confidence Interval.

## Competing interests

The authors declare that they have no competing interests.

## Authors' contributions

STT: was responsible for the design of the research, collection of data, analysis and helps write-up. AM conducted the analyses and write-up of manuscript. AMH helped with the design, data collection and write-up. MHF helped with the data collection and write-up. TN conducted the data collection and helped with the write-up. MBA helped with the design and final write-up of this paper.
